# Smartphone-Based Self-Assessment of Stress in Healthy Adult Individuals: A Systematic Review

**DOI:** 10.2196/jmir.6397

**Published:** 2017-02-13

**Authors:** Helga Þórarinsdóttir, Lars Vedel Kessing, Maria Faurholt-Jepsen

**Affiliations:** ^1^ Psychiatric Center Copenhagen, Rigshospitalet, Department O Copenhagen Denmark

**Keywords:** smartphone, emotional stress, healthy individuals, self-report, objective smartphone generated measures of stress

## Abstract

**Background:**

Stress is a common experience in today’s society. Smartphone ownership is widespread, and smartphones can be used to monitor health and well-being. Smartphone-based self-assessment of stress can be done in naturalistic settings and may potentially reflect real-time stress level.

**Objective:**

The objectives of this systematic review were to evaluate (1) the use of smartphones to measure self-assessed stress in healthy adult individuals, (2) the validity of smartphone-based self-assessed stress compared with validated stress scales, and (3) the association between smartphone-based self-assessed stress and smartphone generated objective data.

**Methods:**

A systematic review of the scientific literature was reported and conducted according to the Preferred Reporting Items for Systematic Reviews and Meta-Analysis (PRISMA) statement. The scientific databases PubMed, PsycINFO, Embase, IEEE, and ACM were searched and supplemented by a hand search of reference lists. The databases were searched for original studies involving healthy individuals older than 18 years, measuring self-assessed stress using smartphones.

**Results:**

A total of 35 published articles comprising 1464 individuals were included for review. According to the objectives, (1) study designs were heterogeneous, and smartphone-based self-assessed stress was measured using various methods (e.g., dichotomized questions on stress, yes or no; Likert scales on stress; and questionnaires); (2) the validity of smartphone-based self-assessed stress compared with validated stress scales was investigated in 3 studies, and of these, only 1 study found a moderate statistically significant positive correlation (*r*=.4; *P*<.05); and (3) in exploratory analyses, smartphone-based self-assessed stress was found to correlate with some of the reported smartphone generated objective data, including voice features and data on activity and phone usage.

**Conclusions:**

Smartphones are being used to measure self-assessed stress in different contexts. The evidence of the validity of smartphone-based self-assessed stress is limited and should be investigated further. Smartphone generated objective data can potentially be used to monitor, predict, and reduce stress levels.

## Introduction

Many people experience stress, in one form or another, throughout their lives. Stress can be defined as “a state, which is accompanied by physical, psychological or social complaints or dysfunctions and which results from individuals feeling unable to bridge a gap with the requirements or expectations placed on them” [1]. Overall, stress can be divided into 2 types: acute and chronic. Acute stress results from a specific event or situation, is short-lived, and can be accompanied by physical symptoms such as a quickening heartbeat, sweating, and headaches, but can also create motivation to deal with whatever is causing the stress. Chronic stress is the response to prolonged pressure and can stem from traumatic experiences or from the wear and tear of daily stress over a longer time period [2]. Work is the most common cause of stress in the Western world, and more than 1 in every 5 European workers feel stressed [3], whereas 65% of Americans state that they are stressed because of their work [4]. Chronic stress causes overexposure of the body to cortisol and other stress hormones and can be a risk factor for developing diseases. Chronic stress has been associated with cardiovascular problems [5], gastrointestinal problems [6], depression [7], and other psychiatric illnesses [8].

People suffering from chronic stress may be less likely to notice whether they are under high stress at a given time point. Using self-assessment of stress during a time period could potentially increase awareness of stressors and encourage behavioral changes.

In 2015, there were 3.4 billion smartphone subscriptions in the world [9], and it has been estimated that by the year of 2017, one-third of the world’s population will use a smartphone [10]. Smartphones can be used for communication, banking, games, looking up information on the Internet, and so forth. During recent years, there has been a growth in the use of smartphones for health monitoring; a search for “health monitor” in Apple’s app store alone yields more than 350 results. Smartphone apps can be used to monitor physical activity, calorie intake, sleep quality, the menstrual cycle, and other issues related to health and well-being [11]. Furthermore, monitoring can take place automatically through the sensors embedded within the smartphone, such as accelerometer and microphone, whereas others require that the users interact with the app to register data [12].

Subjective self-assessed stress can be measured using smartphones via ecological momentary assessment (EMA). EMA is a collection of methods used to collect “assessments of subjects’ current or recent states, sampled repeatedly over time, in their natural environment” [13]. Advantages of using EMA such as minimization of recall bias [14] and collection of fine-grained real-life data collected during non-laboratory settings have been addressed [15]. Subjective stress can be assessed throughout the day using a time-based EMA where people are prompted to rate or answer questions about their “current stress level” [16]. During recent years, the use of smartphones has been explored within bipolar disorder [17-19], depression [20], and anxiety [21].

Many people carry their smartphones with them throughout the day and are used to interacting with it in many locations, in many situations, and at all times [22]. Thus, smartphone-based data could potentially reflect a person’s real-time stress level. Combining smartphone-based self-assessed stress measured by EMA with other smartphone data could help to understand stress better, both on an individual level and on a group level.

However, with no systematic review within this area, the extent to which smartphone-based self-assessed stress has been monitored and evaluated in healthy individuals is unknown. Furthermore, the validity of smartphone-based self-assessed stress compared with other validated stress scales has not been evaluated systematically. Thus, the objectives of this systematic review were to evaluate (1) the use of smartphones to measure self-assessed stress in healthy adult individuals, (2) the validity of smartphone-based self-assessed stress compared with validated stress scales, and (3) the association between smartphone-based self-assessed stress and smartphone generated objective data.

This was the first systematic review of smartphone-based self-assessed stress in healthy adult individuals.

## Methods

### Overview

This systematic review was conducted and reported according to the Preferred Reporting Items for Systematic Reviews and Meta-Analysis (PRISMA) statement [23]. Methods of the review process and eligibility criteria were established in advance and documented in a review protocol that can be retrieved from the authors upon request. No changes were made to the protocol during the review process.

### Eligibility Criteria

Original studies involving healthy individuals older than 18 years measuring self-assessed stress on a smartphone were eligible for review. The language of publication was restricted to English. Papers not meeting eligibility criteria or only describing the technical part of the self-assessment of stress were excluded from review. Where multiple articles were reported on the same study, the article presenting the largest and most detailed dataset was included for review. Only studies in which self-assessed stress was reported on smartphones were eligible for review.

### Information Sources and Search Strategy

Published studies were identified by conducting a systematic literature search through the electronic databases PubMed, PsycINFO, Embase, IEEE, and ACM. The literature search was supplemented by a hand search of reference lists of retrieved articles. The literature search was conducted by 1 researcher (HP), without time restrictions, using the following keywords: (stress or psychological stress or emotional stress) AND (smartphone or cell phone or cellular phone or mobile phone or mobile application or ecological momentary assessment or experience sampling method) and covered a period from 1980 to May 2016. The last literature search was conducted on May 4, 2016.

### Study Selection and Data Extraction

**Figure 1:**
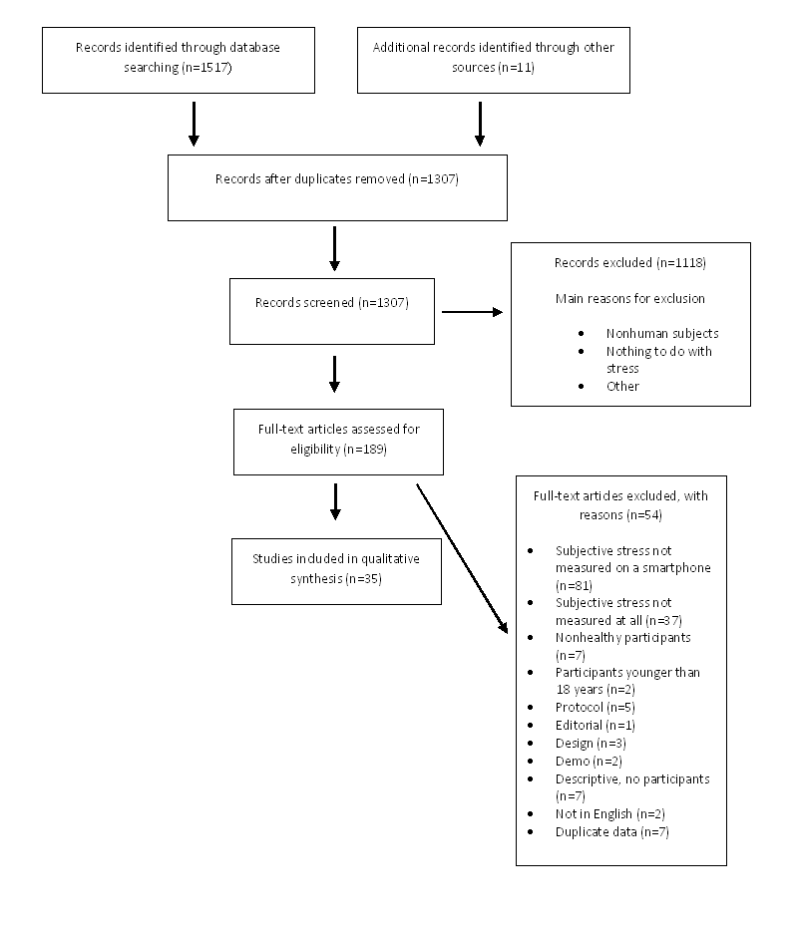
Flow diagram of literature search according to Preferred Reporting Items for Systematic Reviews and Meta-Analyses (PRISMA).

A PRISMA flow diagram of the study selection process is presented in Figure 1. All identified titles and abstracts were screened for eligibility by 1 researcher (HP). Potentially relevant articles were retrieved and full-text articles then checked for fulfilling eligibility independently by 2 researchers (HP and MFJ). One researcher extracted data (HP), and a second reviewer (MFJ) independently checked the extracted data. Any disagreements were resolved by a discussion between 3 researchers (HP, MFJ, and LVK).

## Results

### Study Selection

The literature search identified a total of 1517 articles from the 5 databases, and 11 additional studies were identified by hand search of reference lists. Removing duplicates left 1307 articles for further evaluation. Reviewing abstracts and titles resulted in the exclusion of a total of 1118 articles for not meeting eligibility criteria, the 2 main reasons for exclusion being not including human subjects and not involving stress. Thus, 189 full-text articles were evaluated for eligibility. Of these, 154 articles were excluded from the review for various reasons (Figure 1), with the main reasons being (1) subjective stress not measured on a smartphone (n=81) and (2) subjective stress not measured at all (n=37). A list of excluded articles can be retrieved from the authors upon request. Thus, a total of 35 articles fulfilled the eligibility criteria and were included for review [24-58].

### Study Characteristics

Of the 35 studies, 17 were from the United States and the remainder were from Finland (n=3), Italy (n=4), Germany (n=2), Switzerland (n=2), the United Kingdom (n=3), Australia (n=1), Hong Kong (n=1), Portugal (n=1), and Sweden (n=1). The majority of studies were prospective observational studies [24,28,29,31,33-38,41-45,49-58], 2 were randomized control trials [39,47], 4 were other types of intervention studies [27,40,46,48], 2 were case reports [25,26], and 1 was a cross-sectional study [32]. The study period ranged from 1 hour to 191 days. All the studies were published recently, with the oldest one published in 2007 [44] and more than half of the studies published since 2013 [24,25,27,29-34,36,37,40,42,45,47-54,56-58]. More than half of the studies were published in conference proceedings (n=19), whereas 16 studies were published in peer-reviewed scientific journals (Table 1).

**Table 1:** Characteristics of studies on smartphone-based self-assessed stress in healthy adult individuals included for systematic review (Studies: N=35).

Author	Publication year	Publication type	Study design	Study location	Study duration (days)	Number of participants, context of assessment	Method for self-assessment of stress	Times per day stress measured	Smartphone operating system
Adams et al [24]	2010	Conference paper	Cohort	United States	10	7, daily life	Taylor 5-item measure	Multiple	Android
Atz [25]	2013	Journal article	Case report	United Kingdom	56	1, daily life	7-point Likert scale	Multiple	iOS
Ayzenberg et al [26]	2012	Conference paper	Case report	United States	8.3	1, daily life	7-point Likert scale	N/A	N/A
Bandiera et al [27]	2016	Journal article	Interventional	United States	14	139, smoking cessation	5-point Likert scale	5	Android
Berndt et al [28]	2011	Conference paper	Cohort	Germany	1	50, daily life	0-100 scale	Multiple	N/A
Bogomolov et al [29]	2014	Conference paper	Cohort	United States	190	117, daily life	7-point scale	1	Android
Carroll et al [30]	2013	Conference paper	Cohort	United States	4	12, emotional eating	Russel Circumplex model	Multiple	Windows
Ceja et al [31]	2015	Journal article	Cohort	Italy	40	30, workplace stress	5-point scale	3	Android
Ciman et al [32]	2015	Conference paper	Cross-sectional	Switzerland	0.04 (1 hour)	13, laboratory	5-point Likert scale	N/A	Android
Ferdous et al [33]	2015	Conference paper	Cohort	Italy	42	28, workplace stress	5-point scale	3	Android
Gaggioli et al [34]	2011	Journal article	Cohort	Italy	7	6, daily life	10-point Likert scale	Multiple	Windows
Gomes et al [35]	2012	Conference paper	Cohort	Portugal	191	5, workplace stress	Questionnaire	N/A	Android
Huang et al [36]	2015	Conference paper	Cohort	United States	28	14, daily life	N/A^a^	N/A	Android
Huh et al [37]	2014	Journal article	Cohort	United States	7	26, smoking behavior	Perceived stress	5	Android
Jin et al [38]	2012	Conference paper	Cohort	Hong Kong	2	30, workplace stress	N/A	Multiple	Android
Kennedy et al [39]	2011	Journal article	Interventional	United Kingdom	33	198, vitamin intake	VAS^b^	2	Other
Lachmann et al [40]	2016	Journal article	Interventional	Sweden	14	33, interprofessional learning	7-point Likert scale	5	N/A
Madan et al [41]	2010	Conference paper	Cohort	United States	73	70, epidemiology	Yes or no	1	Windows
Muaremi et al [42]	2013	Journal article	Cohort	Switzerland	112	35, workplace stress	Continuous response value	5	iOS
Muukkonen et al [43]	2008	Conference paper	Cohort	Finland	14	55, studying	Yes or no	5	Symbian
Muukkonen et al [44]	2007	Conference paper	Cohort	Finland	14	8, studying	Yes or no	5	Symbian
Ottaviani et al [45]	2015	Journal article	Cohort	Italy	1	42, daily life	Yes or no	Multiple	Android
Parkka et al [46]	2009	Journal article	Interventional	Finland	56	17, workplace stress	Sliding scale	1	Symbian
Pipingas et al [47]	2013	Journal article	Interventional	Australia	112	38, vitamin intake	VAS	0.14 (once a week)	N/A
Reitzel et al [48]	2014	Journal article	Interventional	United States	13	22, smoking cessation	5-point Likert scale	5	Android
Sano et al [49]	2015	Conference paper	Cohort	United States	30	66, daily life	Calmness	2	Android
Sano and Picard [50]	2013	Conference paper	Cohort	United States	5	18, daily life	0-100 scale	2	Android
Sarker et al [51]	2014	Conference paper	Cohort	United States	7	30, daily life	6-point scale	Multiple	N/A
Vhaduri et al [52]	2014	Journal article	Cohort	United States	7	30, driving	6-point Likert scale	Multiple	Android
Wang et al [53]	2014	Conference paper	Cohort	United States	70	48, daily life	Taylor 5-item measure	Multiple	Android
Weppner et al [54]	2013	Conference paper	Cohort	Germany	84	9, daily life	10-point Likert scale	10	Android
Witiewitz et al [55]	2012	Journal article	Cohort	United States	21	86, concurrent drinking and smoking	5-point Likert scale	3	N/A
Wray et al [56]	2015	Journal article	Cohort	United States	14	76, smoking behavior	5-point Likert scale	4	iOS
Zenk et al [57]	2014	Journal article	Cohort	United States	7	100, snack-food intake	Yes or no	5	Android
Zenonos et al [58]	2016	Conference paper	Cohort	United Kingdom	11	4, workplace stress	0-100 scale	Multiple	Android

^a^N/A: not available.

^b^VAS: visual analog scale.

### Study Participants

Overall, the studies comprised a total of 1464 healthy adult participants, with sample sizes in individual studies varying from 1 to 198 participants. The mean age of the participants was available for 19 of the studies and ranged from 20.1-52.47 years [27,31-34,37-39,43-46,48-52,55,57]. Gender distribution was available for 25 studies [24,27,30-34,36,37,39-42,45-53,55-57], and of these, 4 studies had equal gender distribution [34,47,51,52], whereas there were 2 large gender-specific studies, 1 male [39] and 1 female [57]. In 9 of the studies, the participants were exclusively students [36,40,41,43,44,49,51-53,55], and in 4 studies, the participants were exclusively employees [31,33,42,58].

### Smartphones

The majority of the studies used Android-based smartphones (n=19), 3 used Windows-based smartphones [30,34,41], 3 studies used iPhones [25,49,56], 4 studies [39,43,44,46] used other types of smartphones, whereas the remaining 6 studies did not specify what type of smartphones or operating systems were used [26,28,40,47,51,55]. In 15 of the studies, smartphones were provided for the participants, whereas participants used their own smartphones in 4 studies [32,41,42,49]. Although some participants used their own smartphone in 4 studies, other participants borrowed a smartphone [24,37,53,56]. In total, 12 studies did not specify ownership of the smartphones used [25,26,28,30,39,43,44,50,51,55,57,58].

### Self-Assessed Stress

Overall, the included studies used many different methods to measure smartphone-based self-assessed stress. The most common method (n=11) was using a Likert scale (from a 5-point scale to a 10- or 100-point scale) [25-28,32,34,40,48,50,52,54-56,58]. Five studies used a yes or no answer question to measure self-assessed stress [41,43-45,57], and 5 studies used questionnaires [24,49,50,53]. Two studies did not specify how smartphone-based self-assessed stress was measured [36,38].

The frequency of smartphone-based self-assessed stress reports varied *.* In most of the studies, participants were asked to report their stress levels multiple times per day: from twice a day [39,49,50] to up to once every half hour [24]. In 1 study, participants reported self-assessed stress on a weekly basis [47], whereas in 3 studies, self-assessed stress was reported once per day [29,41,46]. In 4 studies, the frequency of self-assessment was not specified [26,32,35,36].

### Context

Six studies investigated self-assessed stress in the context of the workplace [31,33,35,38,42,58], and 1 study in relation to rehabilitation after work-related stress [46]. Two studies measured self-assessed stress in relation to smoking cessation [27,48], 2 in relation to smoking behavior [37,56], and 1 in relation to concurrent smoking and drinking [55]. Two studies investigated self-assessed stress levels in relation to vitamin intake [39,47], 1 in relation to emotional eating [30], and another in relation to snack-food intake [57]. Three studies looked at self-assessed stress in the context of studying [40,43,44]. One study was done in a laboratory context [32], another in relation to driving [52], and a third looked at stress levels in epidemiological behavior context [41]. The remaining studies (n=13) reported no specific context, and participants registered self-assessed stress during their everyday life. About half (n=16) of the studies investigated stress as the primary objective [24-26,28,29,31-33,35,38,42,50,52,54,58].

### Validity of Smartphone-Based Self-Assessed Stress

In 5 studies, validated stress scales in addition to smartphone-based self-assessed stress were reported. Four of these studies used the Perceived Stress Scale (PSS) [24,49,50,53,59], and 1 used Derogatis Stress Profile (DSP) [46,60]. In 2 of the studies, participants filled out the PSS at baseline only [24,50], and in 2 studies, participants filled out the PSS at both baseline and follow-up [49,53]. The study using DSP was an interventional study, and participants filled out the scale 4 times during the study period [46].

Three studies investigated the correlation between smartphone-based self-assessed stress and validated stress scales [24,46,53]. Adams et al reported a statistically nonsignificant correlation (*r*=.562, *P*=.11) between smartphone-based self-assessed stress levels and PSS score [24]. Another study by Parkaa et al reported a statistically nonsignificant correlation (ρ=.07, *P*=.64) between smartphone-based self-assessed stress and DSP score [46]. Finally, a study by Wang et al reported a statistically significant positive moderate correlation between smartphone-based self-assessed stress and PSS score both pre- (*r*=.458, *P*=.003) and poststudy (*r*=.412, *P*=.009) [53].

### Smartphone Generated Objective Data

A total of 13 studies collected smartphone generated objective data [24,26,29,31,33,41,42,48-53]. Six studies investigated the association between smartphone generated objective data and smartphone-based self-assessed stress [24,29,31,33,41,49]. Among these, 2 studies investigated the association between smartphone-based self-assessed stress and verbal data [24,33]; Adams et al reported a statistically positive correlation (*r*=.59, *P* value not specified) between smartphone-based self-assessed stress and voice-stress, whereas Ferdous et al reported a significant positive correlation between smartphone-based self-assessed stress and duration of verbal interaction for 17 of their 28 participants (*r*=.06-.55, *P*<.005).

A study by Madan et al reported that communication diversity was reduced for participants who often assessed themselves as being stressed, and the authors interpreted this as a tendency to isolate [41]. A study by Sano et al reported that higher self-assessed stress levels were statistically significantly correlated with lower activity level in the evening, fewer and shorter text messages sent, and less screen activity in the evening [49].

Two studies investigated the association between self-assessed stress and smartphone generated objective data in order to detect stress [29,31]. A study by Ceja et al looked at smartphone generated objective data from the accelerometer and “achieved a maximum overall accuracy of 71% for user-specific models and an accuracy of 60% for the use of similar-users models” to classify self-assessed stress levels [31]. A study by Bogomolov et al collected both social features (phone calls and text messages) and proximity features (Bluetooth) and obtained “the accuracy score of 72.28% for a 2-class daily stress recognition problem” [29].

## Discussion

### Principal Findings

This was the first systematic review on smartphone-based self-assessment of stress in healthy adult individuals. A total of 35 published articles involving a total of 1464 participants were included for review. Overall, the study designs were highly heterogeneous, using various methods of self-assessment in different contexts. Most of the studies were conducted in the United States or Western Europe. Android-based smartphones were most commonly used for measuring self-assessed stress, many participants borrowed smartphones during the studies, and often stress was reported multiple times per day.

Regarding the validity of smartphone-based self-assessed stress levels, stress levels measured using validated stress scales were available in 5 studies, but only 3 of these studies investigated the correlation between smartphone-based self-assessed stress and validated stress scales. Among these 3 studies, only 1 study found a statistically significant positive correlation between self-assessed stress and a validated stress scale (PSS) [53]. It should be noted that the study by Wang et al included a larger sample (n=48) compared with the other 2 studies combined (n=7; n=17) [24,46], suggesting a low statistical power of the other 2 studies. In addition, the study by Wang et al included university students on a university campus, limiting the generalizability of the study findings. The validity of smartphone-based self-assessment of stress may be different across populations and should be investigated further in future studies. Thus, findings from this systematic review suggest that the validity of smartphone-based self-assessed stress has been sparingly investigated and is unknown. The studies included described convergent validity of smartphone-based self-assessment of stress. Other parameters such as sleep, mood, and activity level may correlate with validated stress scales; however, content validity was not investigated in this review. In addition, the reliability and predictive validity of smartphone-based self-assessment of stress were also not investigated.

Smartphone generated objective data were collected in 13 studies and 6 studies investigated the association between smartphone-based self-assessed stress and these objective data. Two studies found a positive correlation between self-assessed stress and verbal data, whereas another 2 studies found associations between self-assessed stress and communication diversity, activity levels, text messages, and screen on or off patterns. The last 2 studies found smartphone generated data to be a predictor (accuracy up to 72.28%) for detecting self-assessed stress. Overall, regarding smartphone generated objective data, the studies collected various smartphone generated data and the results seem exploratory, with a tendency to report statistically significant positive correlations with self-assessed stress only.

A majority of the included studies collected objective data alongside the self-assessed data. Some of them used physiological measures collected from various worn sensors, but others only used objective data collected from sensors embedded within the smartphones. Seven studies collected all 3 kinds of stress measures. Collecting physiological measures such as heart rate requires participants to carry additional sensors (user burden), whereas smartphone generated objective data are collected from a smartphone that is most likely already being carried around. Smartphone generated objective data can usually be collected automatically, eliminating attrition due to monitoring. Objective smartphone data are behavioral data that can reflect behavior related to stress. Different people react differently to stress, and combined with self-assessed data on stress, smartphone generated objective data might be used for detecting stress. Early stress detection in healthy populations such as students and employees could help to prevent stress-related diseases. Thus, the use of smartphone generated objective data as a marker of stress in healthy individuals has been sparingly investigated and future well-designed studies investigating this would be interesting.

Stress levels were assessed from self-reported data, both from smartphones and from validated scales. PSS was developed in 1983. It has 10 questions and is widely used within psychological and psychiatric sciences. It has shown good internal reliability (Cronbach alpha=.78-.91 [61]) and is correlated with various self-report and behavioral criteria [59]. DSP is a 77-item self-report inventory developed in 1980 and has also shown good internal reliability (Cronbach alpha=.83-.88 [62]). It should be emphasized that the different methods for self-assessment of stress, smartphone-based and validated scales, do not necessarily measure the same thing. Validated stress scales measure more long-term stress levels, whereas self-assessment on smartphones is more about current stress levels. Validated scales such as PSS have a somewhat clear definition of stress, as they have several items that the participants have to answer. Many of the smartphone-based self-assessment measures of stress were not explicit in their definition of stress, and participants often only answered 1 question about their level of stress. Stress is a popular term and can mean different things to different people; some people might only register stress that they experience as a negative thing, whereas others might also register the kind of stress (eustress) that is positive and can be motivating. As noted in a study by Muaremi et al, stress was not necessarily a negative event or feeling for some of the participants [42].

Registering self-assessed stress multiple times a day can be a tool to help people self-monitor stress levels. In this way, self-monitoring may play a role in helping people to manage stress. Self-monitoring brings awareness of stress levels and encourages behavioral change according to a situation [63]. However, being asked to self-assess one’s stress level up to multiple times a day could introduce a negativity bias. This could result in participants assessing their stress to be higher than it actually is and even potentially cause more stress per se. It may be that measurements in themselves are stressful, but also the situation to have the self-assessed results of chronic stress constantly at hand and to be unable to cope with a given stressful situation. In this case, people may be constantly reminded that they are unable to cope with stress, which may be the reason they are measuring self-assessed stress in the first place. Investigating the effect of introducing coaching or coping elements to the self-assessment apps would be interesting. It should be stressed that we identified no study that investigated whether the use of smartphone to continuously monitor stress—subjectively reported or objectively assessed—per se had a reducing effect on stress level. Whether self-assessments multiple times a day would be a threat to the reliability and validity is unknown and should be investigated further. Most studies looked at self-assessed stress in everyday life, either without context or in the context of work or studying. Many people carry their smartphones with them during most of the day and therefore smartphones are a device well suited for this type of data collection. Registering stress multiple times a day, in different situations, can shed light on where and when people are experiencing stress.

A study by Wang et al looked at stress in students over a whole semester and revealed how their self-assessed stress level increased as their workload increased, with the peak being during final examinations [53]. Following a group of people prospectively over time could help distinguish between the normal stresses that come and go and the chronic, potentially health-damaging kind of stress. Being aware of chronic stress is the first step toward eradicating or minimizing it.

Most studies measured self-assessed stress on Android-based smartphones, and many participants were provided with smartphones during the study period. Allowing participants to use their own smartphones to collect self-assessment of stress would be the least disruptive for participants, as they are already familiar with the device. Using one’s own smartphone would also be likely to more accurately reflect real life, especially in regards to the objective smartphone data. It is possible that participants did not, in all cases, own smartphones. It is also possible that the study smartphones were specially programmed for the study or that participants’ smartphones were different from the ones that were required for the study.

Smartphones constitute a new and an exciting research tool within psychological well-being and health care. Nevertheless, the majority of the identified studies have been published in proceedings from technological conferences. In general, many of these studies focused primarily on the technical side of the smartphone system, and a number of these did not present data on population characteristics such as age [24,26,28-30,35,36,41,44,53,54,58], gender [26,28,29,35,38,43,44,54,58], or employment status of participants [24-30,32,34,35,38,39,45,48,50,54,56].

### Limitations

Limitations at a study level: Several concerns regarding the individual studies and outcomes limited the overall findings of this study. The included studies had highly heterogeneous designs and used various methods to measure smartphone-based self-assessed stress. In addition, in many cases studies did not include clear descriptions of the recruitment process. The studies included were at risk of selection bias, and at an individual study level, there was a lack of information on potential confounding factors such as age, gender, and educational level, which possibly could have affected self-assessed stress level. A large part of the studies included a relatively small sample of participants and reported unadjusted statistical analyses. Validated stress scales were only used in 5 studies out of the 35 studies included. More than half of the included studies did not investigate stress as their primary objective, and information was therefore limited: only 1 out of the 4 largest studies had stress as their primary objective. In general, studies focusing on stress had fewer participants (mean n=24.7) compared with the studies not focusing on stress (mean n=56.3). Self-assessed stress was investigated in selected groups, often recruited through convenience sampling at a university or a workplace. In many of the studies, participants were provided with a smartphone to use during the study period, and some participants received economic incentives to fill out the self-assessments of stress. The generalizability of these studies was therefore limited, but findings could be relevant for more narrow populations such as university students. Overall, methodological limitations related to study designs, self-assessments of stress, as well as statistical analyses of the included studies were observed. There is a need for studies investigating the use and validity of smartphone-based self-assessed stress in more general populations.

Limitations at a review level: Some limitations to this review should be mentioned. Research using smartphones is expanding, and due to the intersectionality of this research (medicine, psychology, and information technology), studies are being published in very diverse forms and places. Our review shows that many of these kinds of studies are being published in conference proceedings. Therefore, conducting a search strategy that is able to capture all relevant scientific articles is a challenge. The review process was restricting among healthy smartphone users and articles published in English, which might have reduced the global acceptance.

### Perspectives and Implications

Stress has become a major health problem in the Western world. Awareness of one’s own stress level is important, and smartphones are potentially a proper minimally intrusive tool for self-assessment of stress.

Self-assessment of stress using smartphones in everyday life is a step toward stress awareness. Looking at self-reported stress levels in relation to other more objective data from smartphones, such as geolocation and physical activity, could help to further understanding of stress and stress-related behavior. However, well-designed studies using strict methodology investigating the validity of smartphone-based self-assessment of stress are warranted. Future studies should investigate how to validly measure subjective stress using smartphones, which by nature is accurate in time and place, in contrast to a self-reported scale on stress administered once a day or less frequently. They should also collect information on and address possible confounding factors in the statistical analyses. In addition, and of even more paramount importance, they should investigate in a randomized controlled trial whether the use of smartphone to monitor stress—subjectively or objectively assessed—per se has a beneficial or detrimental effect on stress level.

This review included only studies with healthy adult participants. Smartphones can and are also being used to measure self-assessed stress in various patient populations, especially within the mental health field, where stress is a risk factor. However, addressing this aspect was beyond the scope of this review.

### Conclusions

This systematic review identified 35 studies using smartphones to measure self-assessed stress in healthy adults. The studies were from different countries and used different self-assessment methods in varying contexts, such as in the workplace, in relation to smoking cessation, and on university campuses. Android-based smartphones were most commonly being used, and the validity of smartphone-based self-assessed stress compared with validated stress scales was limited by low statistical power of the individual studies and small number of studies reporting on validated scales. Some smartphone generated objective data, including voice, activity, and general usage data, were associated with self-assessed stress measured on smartphones. Smartphone generated objective data could represent a potential tool for predicting stress levels. There is a need for further studies investigating the validity of smartphone-based self-assessed stress and smartphone generated objective measures of stress using validated stress scales, and studies investigating the beneficial or detrimental effects of smartphone-based monitoring stress, both subjectively and objectively, on stress levels per se.

## Abbreviations

ACM: Association for Computing Machinery EMA: ecological momentary assessment IEEE: Institute of Electrical and Electronics Engineers IT: information technology N/A: not available PRISMA: Preferred Reporting Items for Systematic Reviews and Meta-Analyses
